# Parallel Detection of Refractive Index Changes in a Porous Silicon Microarray Based on Digital Images

**DOI:** 10.3390/s17040750

**Published:** 2017-04-02

**Authors:** Chuanxi Li, Zhenhong Jia, Peng Li, Hao Wen, Guodong Lv, Xiaohui Huang

**Affiliations:** 1 School of Physical Science and Technology, Xinjiang University, Urumqi 830046, China; lcxxju@163.com (C.L.); pl211869@sina.com (P.L.); 2 College of Information Science and Engineering, Xinjiang University, Urumqi 830046, China; hxhdemail@sina.com; 3 The First Affiliated Hospital of Xinjiang Medical University, Urumqi 830054, China; dr.wenhao@163.com (H.W.); lvgdng@163.com (G.L.)

**Keywords:** porous silicon microarray, digital Images, average gray value, refraction index

## Abstract

A new technique for the refractive index change with high-sensitivity measurements was proposed by the digital image of porous silicon (PSi) microarray utilization in this paper. Under the irradiation of a He-Ne laser, the surface images of the PSi array cells with the microcavity structure were obtained by the digital imaging equipment, whereas the refractive index change of each array cells was detected by calculating the average gray value of the image and the refractive index change measurement sensitivity was 10^−4^ order of magnitude. This technique could be utilized in the label-free and parallel detection of refraction index changes induced by a biological reaction in the microarray or the chip.

## 1. Introduction

The biochip (microarray) constitutes a new technology, which is developed from the research and development of the human genome project. It is an effective method for biological information to be obtained efficiently [1,2]. The current high-precision microarray chip structures have included all known human genome sizes [3]. Two types of commercial chip detection methods exist. One method is the confocal scanning detection method based on the photomultiplier tube (PMT), a two-dimensional detection through point-by-point scanning for biochip detection completion. In contrast, it has proven difficult to be popularized due to the corresponding high cost. The other detection method is based on the CCD fluorescence scanner. Both fluorescence intensity and fluorescence position of each reaction point could be collected by a CCD camera, whereas the relevant biological information could be obtained through the relevant software analysis [4,5,6]. Moreover, the detection methods of fluorescent labeling are proven complex and difficult to be labeled, which is time-consuming and laborious, whereas the fluorescent markers are significantly expensive, resulting in high costs. Additionally, due to the introduction of marker molecules, it is possible for the structure and activity of the biological molecules to be affected and changed, usually making the test results difficult to demonstrate the interaction of biological molecules and the authenticity of the characteristics [7,8]. Based on the aforementioned reasons, the non-mark detection technology has gradually become an important development direction in the field of biological analysis in recent years [9,10,11,12,13].

The porous silicon as an excellent biomaterial can be utilized for the preparation of various types of optical biosensors or biochips [14,15]. In recent years, the PSi microarray has been researched and studied all over the world. Syshchyk et al. [16] enhanced the reflectance spectra of multilayer porous silicon for the 33-array detection of human immunoglobulin G to be achieved. In contrast, the experimentation failed to achieve high-throughput chip detection. Xiao et al. [17] and Pei et al. [18] reported the preparation and quantitative analysis of the fluorescent-labeled porous silicon arrays, whereas the fluorescent markers were still utilized. In this paper, a measurement method for the refractive index of the porous silicon microcavity (PSM) microarray is presented and the relationship between the refractive index and the reflectivity of the PSM cell was reported [19].

In this paper, detection of refractive index change in the PSM microarray was implemented by image gray measurement, combining the optical characteristics and digital image processing technology of an array cell PSM structure, whereas a new technique for the rapid biological detection of the label-free array based on the image gray scale was developed.

## 2. Measuring Principle

Each PSM cell of the microarray is a photonic crystal with defect states, consisting of two perfectly symmetrical Bragg reflectors (BR) and an intermediate Fabry-Perot cavity. The optical thickness between the Bragg reflector and the defect layer satisfies the following equations:

*n*_H_d_H_ = *n*_L_d_L_ = *λ*c/4 (1)

**Figure 1:**
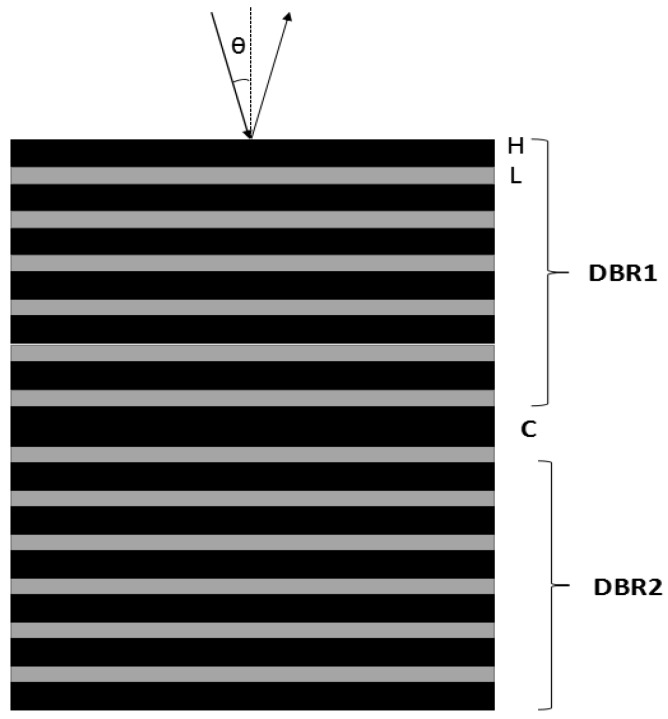
Schematic diagram of PSM structure.

**Figure 2:**
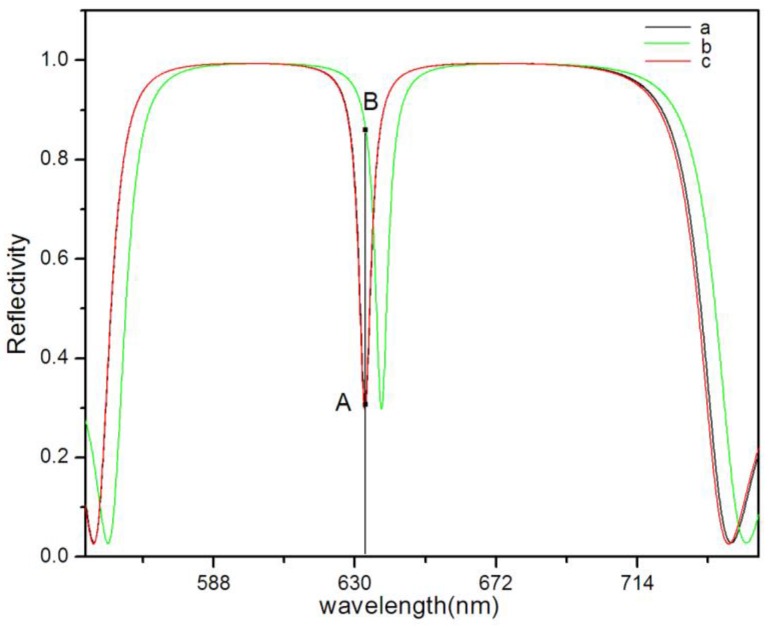
Curve (**a**) represents the reflection spectrum of PSM at normal incidence; curve (**b**) represents the reflection spectrum of PSM which the refractive index increased by 0.01 at normal incidence; curve (**c**) represents the reflection spectrum of PSM which the refractive index increased by 0.01 when the incident angle is 9°.

*n*_C_d_C_ = *λ*c/2 (2)

where *n*_H_, *n*_L_, and *n*_C_ represent the refractive index of the high and low refractive index layer and the defect layer of porous silicon, respectively; the thickness is d_L_ = 100 nm, d_H_ = 140 nm, d_C_ = 560 nm, respectively, and the defect state wavelength is *λ*c = 633 nm. A schematic diagram of the structure is shown in Figure 1. Regarding the vertical incident light (θ = 0°), the theoretical wavelength value of the defect state is *λ*c = 633 nm through the transfer-matrix method. The refractive index of each layer was increased after the biological reaction occurred in the PSM (supposedly the refractive index was increased by 0.01), whereas the calculated value of the defect state wavelength was 638 nm by the transfer matrix method, the reflection spectrum redshift occurred, which is presented as curves a and b in Figure 2. By the incident angle increase, the blueshift of the reflection spectrum would occur. From the calculation, the wavelength of the defect state turned blue to 633 nm if the incident angle increased to 9° and the reflection spectrum c with curve a almost coincided [20,21,22].

**Figure 3:**
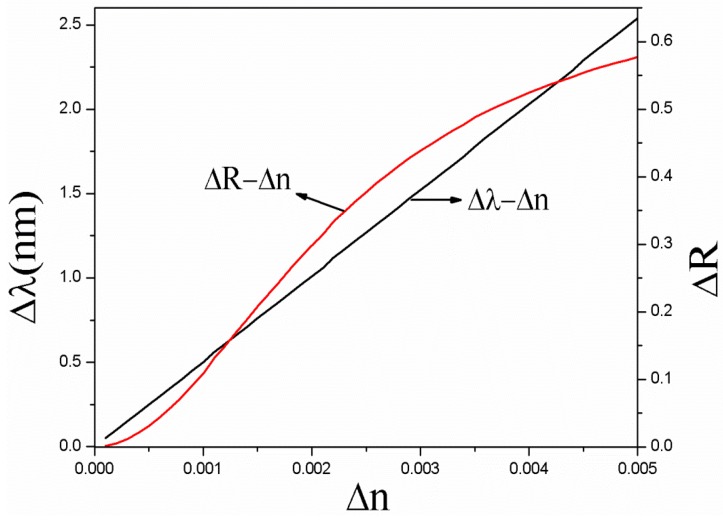
Relationship among the three Δn, ΔR, and Δλ.

The defect state wavelength λc of PSM is designed at 633 nm, for incident light with a wavelength of 633 nm, the reflectivity is 0.3 (point A on curve a or c in Figure 2). If the refractive index of the PSM is increased by 0.01, the λ_c_ of PSM will become 638 nm. In this case, for incident light with a wavelength of 633 nm, the reflectivity is 0.85 (point B on curve b in Figure 2). We analyzed the relationship between the reflectivity change ΔR, the defect state wavelength change Δλ, and the refractive index change Δn by the transfer matrix method. Figure 3 shows ΔR and Δλ increase with the increase of Δn, respectively.

The weakest reflected light would occur if the PSM microarray cell with the defect wavelength of *λ*_C_ = 633 was a normal incident from the laser. If the refractive index of the PSM microarray was increased due to the biological reaction and the defect wavelength in the PSM cell increased, the reflection of 633 nm would be enhanced. The incident angle increase of the laser Δθ could lead the reflected light back to being the weakest. The refractive index change of PSM through the Δθ value calculation constitutes the angle measurement mechanism.

**Figure 4:**
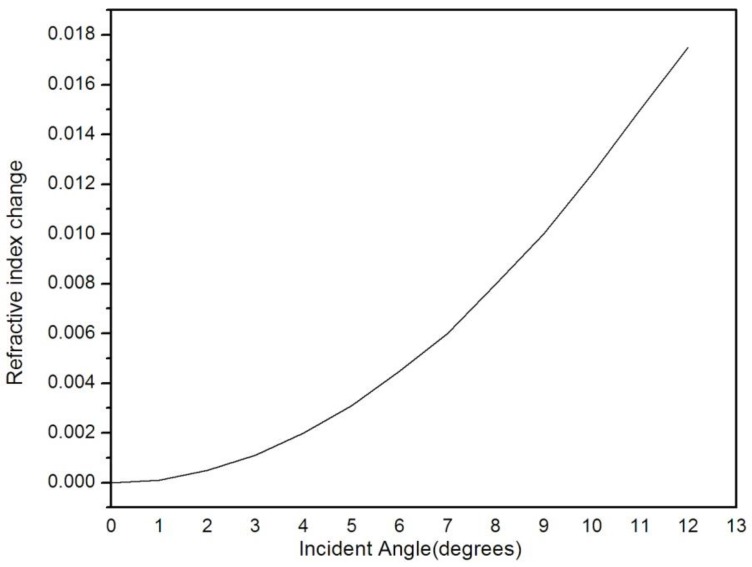
Refraction spectrum of refractive index change versus incident angle.

The change of the PSM refractive index was calculated at the incident angle from 0° to 12° by the transfer matrix method. It could be observed from Figure 4 that, as the refractive index variation increased, the incident angle Δθ increased.

**Figure 5:**
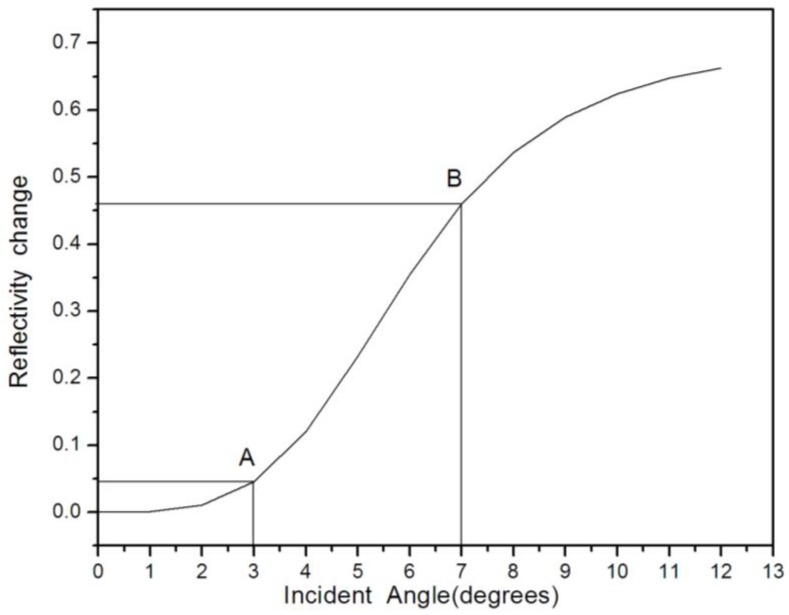
Reflectance spectrum regarding refractive index variation versus incident angle.

**Figure 6:**
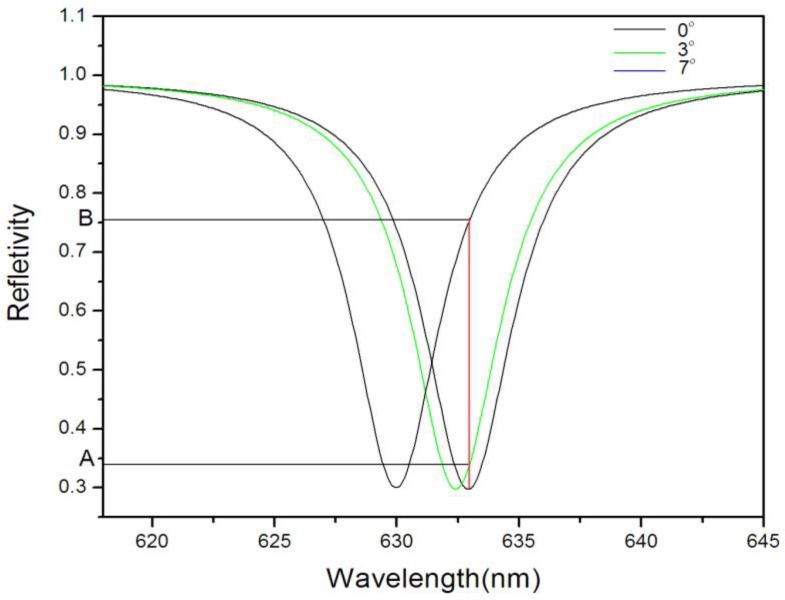
Reflection spectrum versus reflectivity wavelength at incidence angles of 0°, 3°, and 7°, respectively.

Figure 5 demonstrates the spectrum that the transfer matrix method utilized in the reflectivity variation versus the incidence angle change, as the incident angle increased, the change of reflectivity also increased. The change of reflectivity would increase slowly if the incident angle was lower than 3° or higher than 7°. It could be observed from the curve that the curve was approximately linear in the AB segment and the corresponding angles of incidence were 3° and 7°, respectively. Figure 6 presents the reflection spectrum versus the change of wavelength, whereas the incident angles were 0°, 3°, and 7°, respectively. As the incident angle increased, the reflectivity continuously increased, whereas the corresponding reflectivity in the figure between A and B corresponded to the AB segment in Figure 3. Therefore, the reflectivity between 3° to 7° was almost linear with respect to Δθ.

The aforementioned analysis demonstrated that Δθ, Δn, ΔR, and Δλ were in one-to-one correspondence. The biological reaction in PSM results in the change of effective refractive index of PSM. Changing the incident angle of the laser can also affect the reflectivity of PSM. Therefore, by analyzing the influence of the incident angle on the reflectivity, we can know the influence of the refractive index change on the reflectivity.

In this paper, the image gray level method was utilized in the refractive index detection of the PSM microarray, meaning that a single wavelength lambda λ_i_ visible laser was utilized to illuminate a PSM microarray with the defect wavelength lambda λ_i_. When the laser was perpendicular to the PSM array, the reflected light was the weakest. The entire area of the array was imaged by a digital microscope and image processing software (such as MATLAB software) was utilized in the image processing of the central region in one of the arrays. The average gray value of the image increased as the reflectivity increased and the refractive index change could be obtained by the average gray value calculation of the image.

## 3. Experiment

### 3.1. Fabrication of PSM

**Figure 7:**
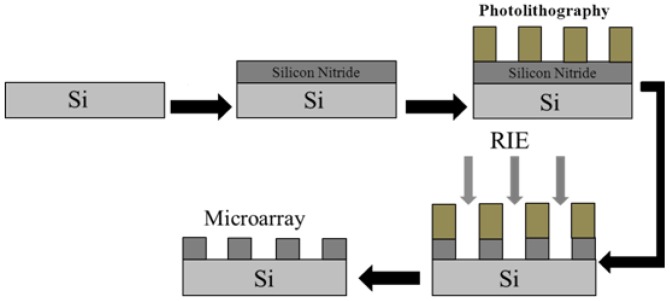
Technological process for preparing the microarray.

**Figure 8:**
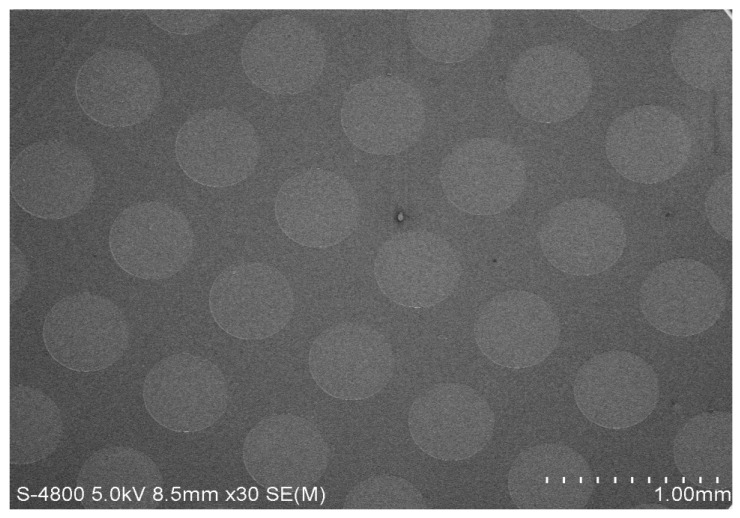
Microarray surface SEM image.

The silicon substrate was a P-type crystalline silicon (resistivity of 0.03–0.06 Ω·cm, crystal orientation of <100>, and thickness of 400 ± 10 μm). The silicon nitride film, with a thickness of 1.5 μm, was deposited on the silicon substrate by plasma-enhanced chemical vapor deposition (PECVD). In the electrochemical etching process, silicon nitride film, as a masking material on silicon, has good etching resistance and, in the preparation of the PS process, can be used to prevent the corrosion of porous silicon within 3–4 min. In order to enhance the stickiness between the silicon nitride layer and the photoresist, we first apply a layer of hexamethyl-disilazane (HMDS) in the silicon nitride film and then coat the HMDS with a 1.1 μm thickness photoresist, and use a mask plate (9 × 9 arrays, each cell has a diameter of 500 μm and spacing of 200 μm) with the array surface pattern to be exposed. A reactive ion etching machine (RIE) was used for etching and was washed repeatedly with acetone. The excess photoresist was removed, and the array was finally dried at room temperature [19,23]. The photolithographic preparation process is shown in Figure 7. The SEM image of the obtained microarray is shown in Figure 8.

The porous silicon microcavities were prepared by the single-channel anode electrochemical etching method, whereas the structure of the electrolytic corrosion was the polytetrafluoroethylene single channel, the copper was the cathode, and the silicon was the anode. The electrolytic etching solution consisted of hydrogen fluoride acid (concentration is 40%) and anhydrous ethanol (C_2_H_5_OH, concentration ≥ 99%) with a volume ratio of 1:1. The microarray was cleaned prior to the experiment: by acetone, ethanol, and deionized water, respectively, under 15 min ultrasounic conditions, for the surface impurities to be removed.

**Figure 9:**
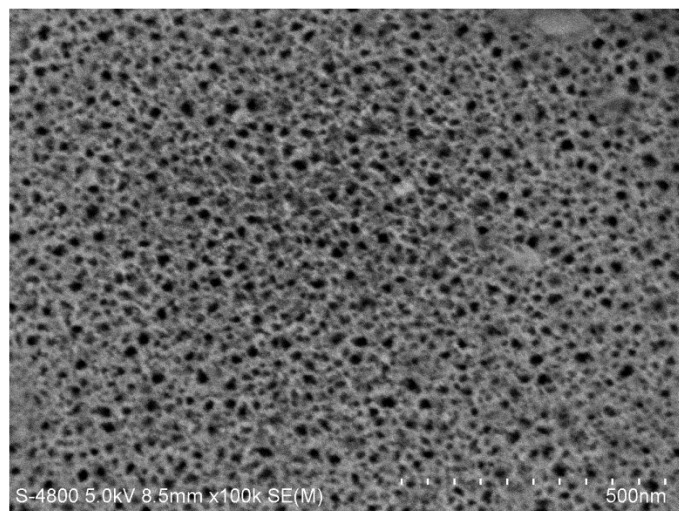
SEM image of PSM microarray single cell surface.

**Figure 10:**
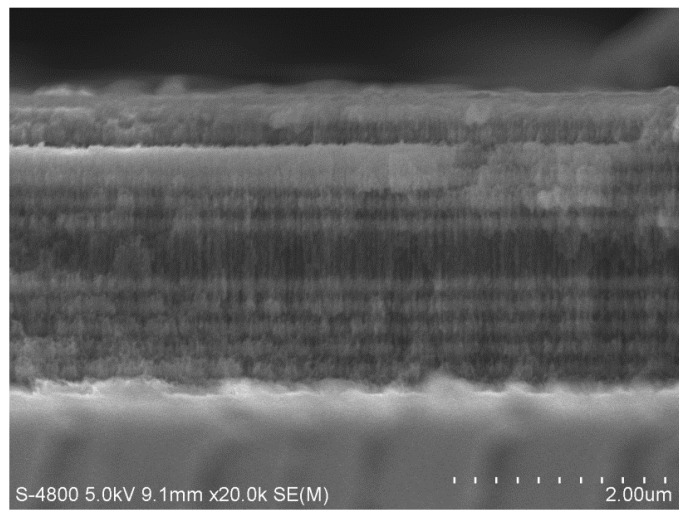
SEM image of PSM microarray single cell cross-section.

The sample electrochemical etching was controlled by Labview software and corroded at room temperature in a dark environment. A current density of 110 mA/cm^2^ and an etching duration of 1.0 s was applied to obtain a low refractive index layer (*n*_L_ = 1.13) with a thickness of 140 nm. A current density of 60 mA/cm^2^ and an etching duration of 1.2 s was applied to obtain a high refractive index layer (*n*_H_ = 1.58) with a thickness of 100 nm. The current density and the etching duration were set to 110 mA/cm^2^ and 2 s for a defect layer with a thickness of 560 nm to be obtained. A pause of 3 s following each layer corrosion should exist in order to ensure relative uniform corrosion for each layer. A total of 25 dielectric layers were etched, and the defect wavelength of PSM was 633 nm. After electrochemical etching, all of the cells in the array (the circular regions) form the PSM, and in other areas outside of these circular regions, the silicon nitride film with a thickness of about 1 μm is covered. Figure 9 presents the SEM micrographs of PSM microarrays, and the pore size of the PSi was approximately 30 nm. Figure 10 presents a SEM image of one array cell of the PSM microarray.

### 3.2. Detection of the PSM Microarray

**Figure 11:**
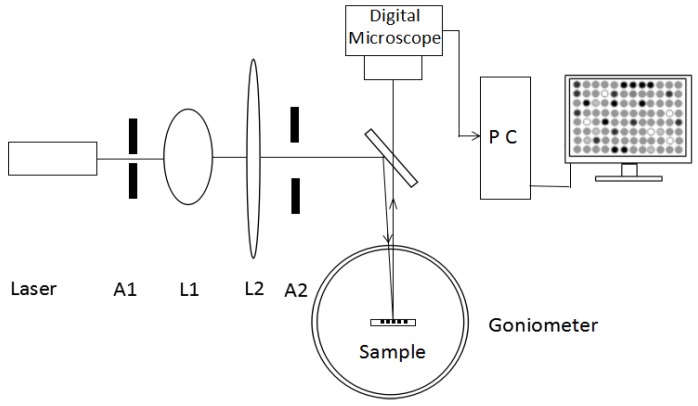
Multiple PSM microarray surface optical properties detection.

Figure 11 demonstrates the optical properties detection of the multiple PSM microarray surface. The light source was a He-Ne laser (λ = 633 nm, 1.8 mW). A1 and A2 were the apertures, and lens L1 and L2 were produced from a collimation beam expander system. The expanded beam passed through a beam splitter (5:5) onto a PSM microarray sample placed at the center of the goniometer. The sample could be rotated with the goniometer and the incident light was reflected to the digital microscope.

**Figure 12:**
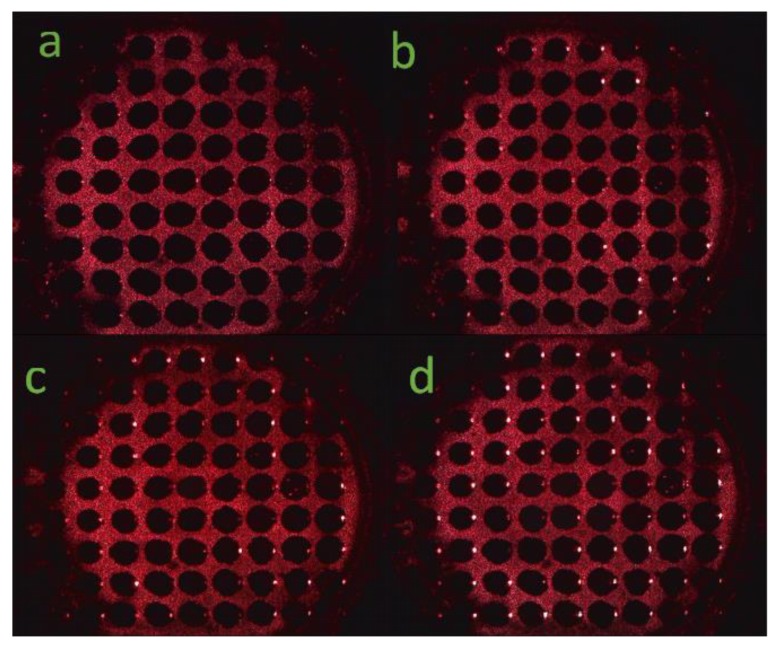
(**a**) Array surface with incident angle of 0°; (**b**) Array surface with incident angle of 3°; (**c**) Array surface with incident angle of 5°; (**d**) Array surface with incident angle of 7°.

During the experiment, the digital microscope received the reflected light from the surface of the array by a beam splitter. As presented in Figure 12a–d, respectively, the corresponding incidence angles were 0°, 3°, 5°, and 7°. It could be observed from Figure 11 that as the incident angle increased, the array surface reflection intensity also increased.

**Figure 13:**
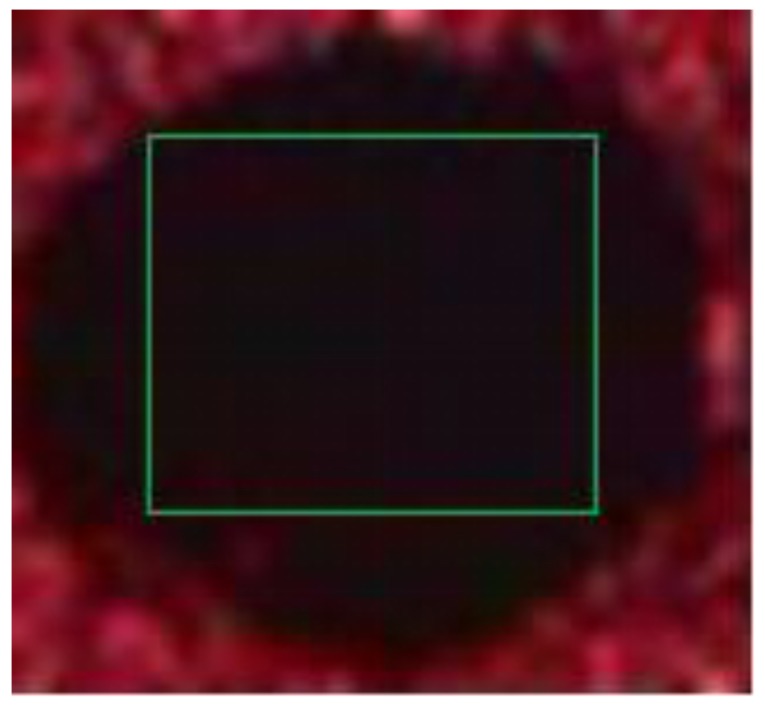
Array surface with incident angle of 1°, the green rectangle area is the average gray value location.

Since the scattered light was formed on the porous silicon rough surface by the incident laser, these coherent scattering lights interfered with each other forming the speckle noise on the CCD surface in the digital microscope. During the image analysis, the image processing software was utilized in the average gray value calculation of one array cell. In Figure 13, an image of one array cell with an incident angle of 1° is presented.

**Figure 14:**
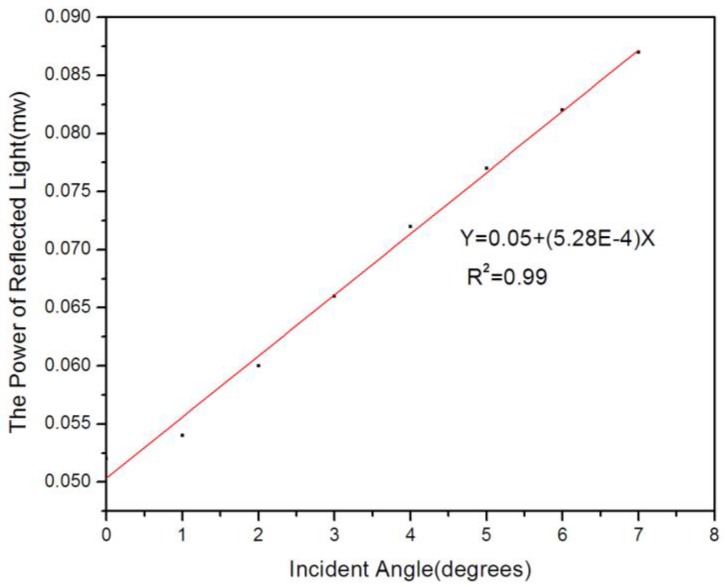
Reflected light of a cell in the array versus the angle.

In the experiment, the reflected light intensity of a cell in the array versus the angle was measured. As a result, presented in Figure 14, it could be observed that the intensity of the reflectance increased as the incident angle increased and both demonstrated a linear relationship.

## 4. Results and Discussion

**Figure 15:**
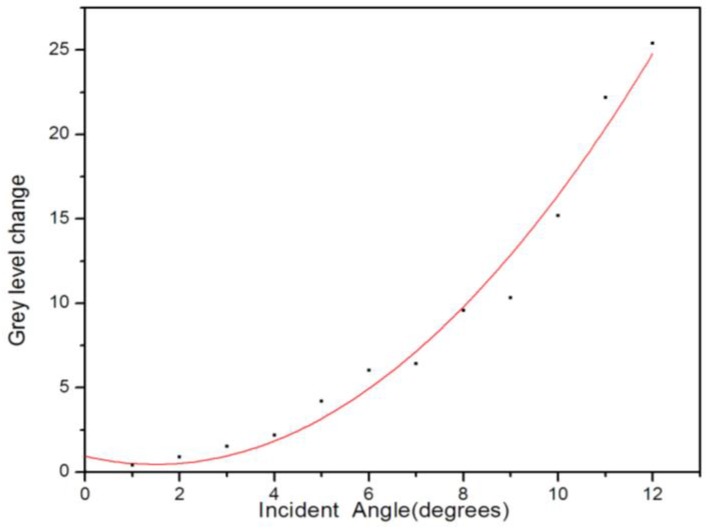
Gray value spectrum of average gray value variation change versus the angle.

The average gray values of the PSM microarray cells from the incident angle of 0° to 12° by the image method were calculated. The angle change led the wavelength of the defect state to a blueshift, which led to the reflectivity and corresponding gray value increase. According to the experimental results in Figure 15, the image average gray value change also increased as the incident angle increased.

**Figure 16:**
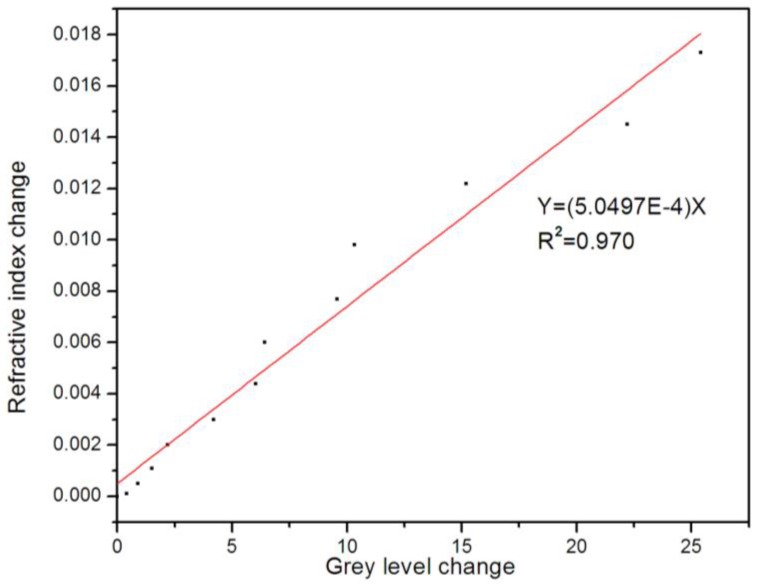
Relationship between refractive index change and gray value in the range of 0°–12°.

The experimental results demonstrated that the refractive index of PSM microarray could be detected by the image method. The gray value change corresponded to the refractive index change. Figure 16 presents the refractive index change variation versus the incident angle from 0° to 12° as the gray value increased displaying a linear relationship. The Y was the theoretical simulation of the refractive index change versus the incident angle from 0° to 12°, whereas the X represented the experimental results of the average gray value versus the angle from 0° to 12°. In the angle range of 0° to 12°, an excellent linear relationship between the change of the refractive index and the change of average gray value (R = 0.970) existed.

Based on the aforementioned results, it could be calculated that if the average gray value change of the array cell was 1, the refractive index change would be 0.00051. The refractive index change of the PSM microarray measurement by image gray value detection had high detection sensitivity, whereas the refractive index change of a 10^−4^ order of magnitude could be detected.

**Figure 17:**
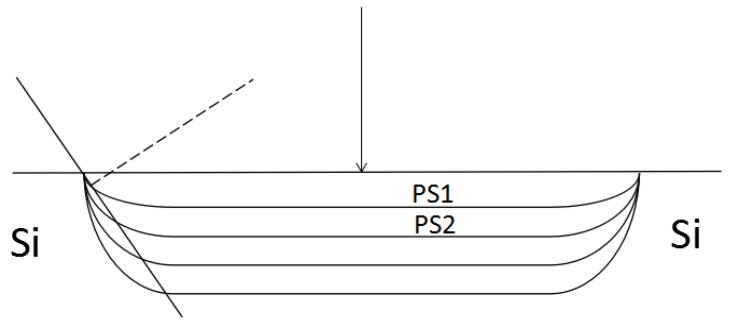
Surface optical path when the laser illuminates PSM microarray vertically.

During the PSM microarray preparation, the electrochemical anodic oxidation method was adopted. At each edge of the each array cell of the PSM microarray, each layer of porous silicon formed a certain tilt, as presented in Figure 17. If the vertical incident laser wavelength was consistent with the PSM defect state wavelength of the array cell, the middle region of the array cell would be dark. The edge region would be equivalent to the oblique incidence of the laser, therefore, the brightness would increase. As the laser beam incidence angle increased, the bright spots appeared on the array cell edge, as presented in Figure 12d, by the right side of each array cell.

The laser power fluctuation effect caused by laser instability on the measurement results could be eliminated by the image gray scale comparison of the array cells that had no refractive index change. Therefore, by the digital image gray value change analysis, the high sensitivity detection of the biological microarray based on the refractive index change could be obtained. Moreover, this type of detection was label-free and parallel.

## 5. Conclusions

In this paper, a new method of the refractive index change measurement of a PSM microarray based on image gray level change detection was proposed. In the experiment, the refractive index change in the array cell could be obtained by the laser incident angle adjustment. The array surface was imaged by a digital microscope, whereas the average gray value was calculated by image processing software. The refractive index changes of the microarray cells were obtained by the average gray value change measurement of each microarray cell. This refractive index change-detecting method has a sensitivity of 10^−4^ order of magnitude, which could be utilized in label-free, fast, parallel, and high-sensitivity biological detection.
